# Eosinophilic Granulomatosis With Polyangiitis Presenting With Purpura, Mononeuritis Multiplex, and Cerebral Infarction

**DOI:** 10.7759/cureus.56949

**Published:** 2024-03-26

**Authors:** Aswinishree Bagavandoss, Jency Kurian, Samson O Oyibo, Olugbenro O Akintade

**Affiliations:** 1 Internal Medicine, Peterborough City Hospital, Peterborough, GBR; 2 Diabetes and Endocrinology, Peterborough City Hospital, Peterborough, GBR; 3 Geriatrics, Peterborough City Hospital, Peterborough, GBR

**Keywords:** anca associated vasculitis, cerebral infarction, mononeuritis multiplex, purpura, eosinophilic granulomatosis with polyangiitis (egpa)

## Abstract

We present a case of a 52-year-old woman who had transient speech impediment and progressive numbness, weakness, and a purpuric rash affecting her limbs, with severe joint pains. Because she had a chest infection two weeks prior, her clinical presentation gave rise to a suspicion of post-infective vasculitis or post-infective polyneuritis. Further investigation proved this to be eosinophilic granulomatosis with polyangiitis (EGPA) presenting with purpura, mononeuritis multiplex, and cerebral infarction. Treatment with glucocorticoids and cyclophosphamide led to rapid remission. This case highlights the potential difficulty in diagnosing EGPA because of its multiple clinical manifestations and emphasizes the importance of a thorough review of the past medical history.

## Introduction

Eosinophilic granulomatosis with polyangiitis (EGPA) is a rare small-vessel vasculitis in patients with asthma and eosinophilia. End-organ involvement includes tissue eosinophilia, necrotizing vasculitis, and eosinophil-rich granulomatous inflammation [1]. The annual incidence of EGPA is 0.9-2.4 per million persons [2]. EGPA usually goes through three phases: a prodromal ‘allergic’ phase, which can last for several years and is marked by asthma and/or chronic rhinosinusitis; an eosinophilic phase, during which eosinophilia and end-organ involvement appear; and a vasculitis phase, due to small- to medium-vessel vasculitis in end-organs (e.g., purpura, mononeuritis multiplex and glomerulonephritis) [3].

The diagnosis of EGPA is very difficult and requires a multidisciplinary team approach to rule out other differential diagnoses and mimics [1]. A thorough review of the past medical history is essential. In the absence of validated diagnostic criteria, a diagnosis of EGPA should be based on highly suggestive clinical features, objective evidence of vasculitis (e.g., histology), and raised myeloperoxidase anti-neutrophil cytoplasmic immunoglobulin-G antibody (pANCA) levels [1,4]. We describe the case of a woman who presented to the emergency department with a combination of neurological, musculoskeletal, and cutaneous symptoms secondary to EGPA.

## Case presentation

Medical history and demographics

A 52-year-old woman presented to the emergency department with a history of transient speech impediment, which lasted a few minutes. She also had an eight-day history of numbness and weakness in both hands and left foot and a transient episode of wrist drop affecting her right upper limb. During the same week, she noticed red blisters over her left hand and behind both knees, accompanied by debilitating joint pains. Two weeks prior to the limb numbness, she had completed a course of clarithromycin for a chest infection. She had no visual or auditory symptoms and no urinary or bowel symptoms. Past medical history included migraine, chronic rhinosinusitis for nine years, and recently discovered bilateral nasal polyps. She did not have asthma. The medication list included fluticasone nasal spray and hormone replacement therapy. She was a non-smoker and drank little alcohol. She had no history of recent travel.

**Figure 1:**
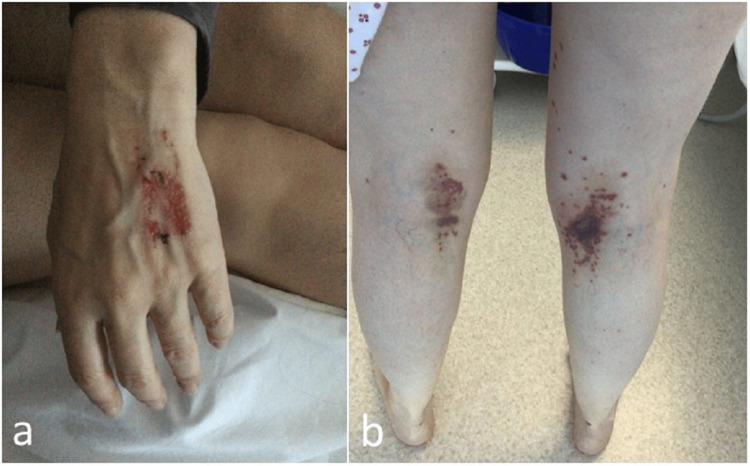
Purpuric rash over left hand and behind both thighs and knees a) Purpuric rash over left hand. b) Purpuric rash behind both thighs and knees

On examination, she had a temperature of 37.3°C, heart rate of 113 beats per minute, respiratory rate of 20 breaths per minute, blood pressure of 138/101 mmHg, and normal oxygen saturation. There was a purpuric rash over her left hand and the posterior aspect of both thighs and knees, suggesting vasculitis (Figure 1). There were no lateralizing neurological signs of a stroke or cranial nerve dysfunction. There were no motor or sensory deficits on examination of her upper and lower limbs, but all her joints exhibited limited movement due to pain and weakness. Chest and abdomen examination were normal.

Investigations

Initial investigations demonstrated marked leukocytosis due to severe eosinophilia, mild thrombocytosis, and a raised C-reactive protein level. Alanine transferase was elevated, but other liver function parameters were normal. The serum immunoglobulin-G was slightly elevated at 16.4 g/L. Renal and thyroid function were normal (Table 1). There was no vitamin B12 or folate deficiency. Screening tests for Coronavirus-19, human immunodeficiency virus (HIV), hepatitis B, and hepatitis C viral infection were negative. Urinalysis was normal. The electrocardiogram and chest x-ray were normal. A lung function test was also normal. CT scan of the brain revealed extensive sinonasal polyposis.

**Table 1:** Initial blood results

Blood test	Result	Reference range
Sodium (mmol/L)	133	132-145
Potassium (mmol/L)	4.1	3.4-5.1
Chloride (mmol/L)	98	97-110
Creatinine ($\mu$mol/L)	50	45-84
Urea (mmol/L)	3.5	2.5-7.8
Calcium (mmol/L)	2.37	2.2-2.6
Phosphate (mmol/L)	1.09	0.8-1.5
Magnesium (mmol/L)	0.73	0.7-1.0
Creatinine phosphokinase (U/L)	56	25-200
25-Hydroxycholecalciferol (nmol/L)	90	50
Thyroid stimulating hormone (mU/L)	3.08	0.3-4.2
C-reactive protein (mg/L)	77	< 5
Estimated glomerular filtration rate (ml/min)	>90	> 60
Total protein (g/L)	71	60-80
Albumin (g/L)	35	35-50
Alanine transferase (U/L)	220	10-60
Alkaline phosphatase (U/L)	126	30-130
Bilirubin ($\mu$mol/L)	9	0-21
Lactate (mmol/L)	2.4	0.6-2.5
Lactate dehydrogenase		
Troponin T (ng/L)	9	<12
Hemoglobin (g/L)	140	115-165
White cell count (10^9^/L)	29.2	4.0-11.0
Eosinophil count (10^9^/L)	14.4	0.1-0.6
Neutrophil count (10^9^/L)	11.9	1.8-7.7
Lymphocyte count (10^9^/L)	2.2	1.4-4.8
Platelet count (10^9^/L)	487	150-400
Prothrombin time ratio	0.98	0.8-1.25
Immunoglobulin G (g/L)	16.4	6.0-16.0
Immunoglobulin A (g/L)	2.71	0.8-4.0
Immunoglobulin M (g/L)	1.01	0.5-2.0
Complement protein C3 (g/L)	1.57	0.75-1.65
Complement protein C4 (g/L)	0.2	0.14-0.54

Subsequent investigation reported an elevated serum myeloperoxidase anti-neutrophil cytoplasmic immunoglobulin-G antibody level (pANCA >134 IU/ml) with normal anti-proteinase-3 level (cANCA < 0.7 IU/ml). Serum electrophoresis was normal, and a test for cryoglobulins was negative. Anti-cardiolipin and anti-beta-2 glycoprotein levels were normal, and a normal dilute Russell viper venom test (DRVVT) was performed.

**Figure 2:**
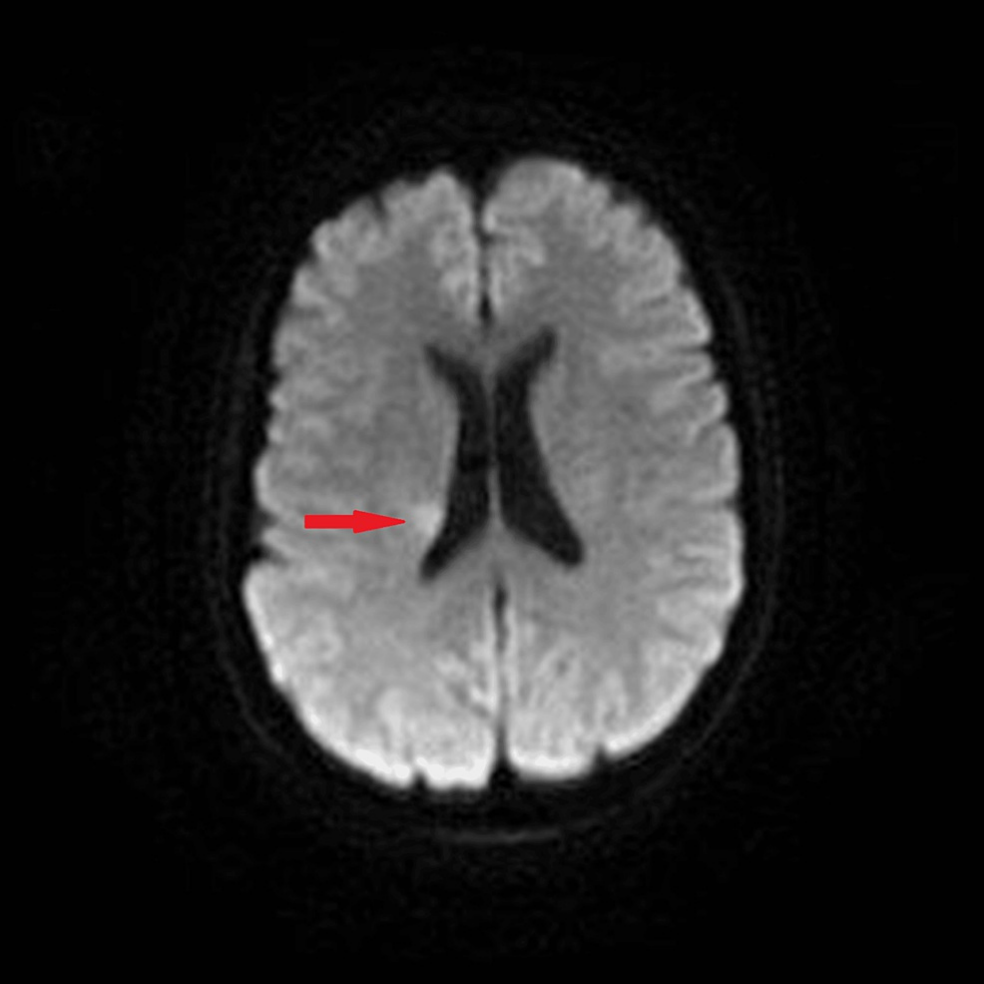
Diffusion-weighted magnetic resonance imaging scan of the brain Axial view demonstrating the subacute infarct in the right corona radiata (red arrow)

Nerve conduction studies reported patchy, asymmetrical sensory and motor abnormalities suggestive of mononeuritis multiplex. Superficial peroneal nerve and peroneal brevis muscle biopsies reported active eosinophilic vasculitis consistent with EGPA. A magnetic resonance imaging scan of the head reported a small subacute infarct in the right corona radiata and small patchy areas of chronic ischemia in the posterior right lentiform nucleus and the right corona radiata more superiorly (we suspected all related to EGPA) (Figure 2). A magnetic resonance imaging scan of the cervical and thoracic spine was reported to be normal.

Treatment

Given the symptoms, the history of chronic rhinosinusitis and recent nasal polyps, the examination finding of mononeuritis multiplex, and the laboratory evidence of severe eosinophilia, a diagnosis of eosinophilic granulomatosis with polyangiitis was suspected. The patient was commenced on 1 g intravenous methylprednisolone daily for 3 days, followed by oral prednisolone. This involved a multidisciplinary team approach (stroke physician, neurologist, and rheumatologist).

Outcome and follow-up

The patient’s symptoms and skin lesions gradually resolved. The patient was discharged after 10 days on a reducing course of oral steroids and later commenced cyclophosphamide therapy. Repeat eosinophil counts, inflammatory markers, alanine transferase, and pANCA levels returned to normal. The patient remained completely well and continued follow-up under the rheumatology and otorhinolaryngology teams.

## Discussion

The patient was initially on the stroke pre-arrival list. However, given a history of recent chest infection, we suspected post-infective vasculitis (Henoch-Schonlein purpura) or post-infective polyneuritis (Guillaine-Barre syndrome). There was no glove-and-stocking paraesthesia characteristic of Guillaine-Barre syndrome, and her lung function studies were normal. There was no renal impairment, making Henoch-Schonlein purpura less likely. The full investigation led us to discover that our patient had EGPA with cutaneous (purpura), central nervous system (subacute cerebral infarct), and peripheral nervous system (mononeuritis multiplex) involvement. This patient had a good clinical outcome because of early diagnosis and treatment by a multidisciplinary team.

Our patient did have a transient rise in the alanine transferase level, which returned to normal during treatment. This may have been due to a transient hepatic inflammation. Additionally, we did not find any neurological features in keeping with the MRI findings, which led us to believe that this patient had asymptomatic cerebral infarcts.

As previously mentioned, there are no diagnostic criteria for EGPA. However, a panel of European experts has developed evidence-based guidelines for the diagnosis and management of EGPA to guide clinical practice and decision-making in EGPA [1].

The American College of Rheumatology/European Alliance of Associations for Rheumatology Classification Criteria for Eosinophilic Granulomatosis with Polyangiitis (ACR/EULAR EGPA) developed a classification system to help differentiate cases of EGPA from similar types of vasculitis in research settings (Table 2) [4]. This classification system only applies when a diagnosis of vasculitis has been made, and alternative diagnoses mimicking vasculitis have been excluded and is not to be used for diagnostic purposes [4]. Such “vasculitis mimics” including Sjogren’s syndrome, rheumatoid arthritis, antiphospholipid syndromes, and infections (e.g., hepatitis, HIV), were excluded in our patient’s case.

**Table 2:** 2022 American College of Rheumatology/European Alliance of Associations for Rheumatology Classification Criteria for Eosinophilic Granulomatosis with Polyangiitis Sum the scores for seven items, if present. A score of > 6 is needed for the classification of EGPA. For research purposes, our patient would have had a score of 11

Criteria	Feature	Score
Clinical criteria	Obstructive airway disease	+3
	Nasal polyps	+3
	Mononeuritis multiplex	+1
Laboratory and biopsy criteria	Blood eosinophil count > 1 x 10^9^/L	+5
	Extravascular eosinophilic-predominant inflammation on biopsy	+2
	Positive test for cytoplasmic anti-neutrophil cytoplasmic antibodies (cANCA) or anti-proteinase 3 (anti- PR3) antibodies	-3
	Hematuria	-1

Guidelines recommend remission-induction treatment based on clinical manifestations with prognostic relevance, using the Five-Factor Score (renal insufficiency, proteinuria, cardiomyopathy, gastrointestinal tract, and central nervous system involvement) [1]. Glucocorticoids should be used to induce remission in severe cases. In patients with unfavorable prognostic factors, cyclophosphamide should be added. In combination with glucocorticoids, Rituximab or mepolizumab can be used for remission maintenance [1].

There are several case reports demonstrating the multiple clinical manifestations of EGPA. In a cohort of 354 patients with EGPA, constitutional symptoms (weight loss, fatigue, arthralgias, myalgias) were the commonest presenting features (58.5%), followed by ear/nose/throat symptoms (56.8%), pulmonary symptoms (55.4%), neurological symptoms (42.7%), cutaneous symptoms (19.2%), cardiac symptoms (14.4%), renal (4.5%) and gastrointestinal symptoms (3.1%) [5]. Our patient had constitutional symptoms, ear/nose/throat pathology, neurological symptoms, and cutaneous symptoms.

## Conclusions

Eosinophilic granulomatosis with polyangiitis (EGPA) has multiple clinical manifestations, making it difficult to diagnose. Several vasculitis mimics must be excluded during the diagnostic work-up. A thorough review of the past medical history and a multidisciplinary approach are crucial for diagnosing and managing EGPA. Given the long prodromal phase, the possibility of yearly rheumatology assessment for patients with a combination of asthma, chronic rhinosinusitis, and nasal polyposis should be considered to prevent the possible onset of vasculitis. Further research is required to prevent progression from the prodromal phase of chronic asthma and chronic rhinosinusitis to the vasculitis phase.
